# Routine Electronic Mother-Infant Data (REMInD): A proof-of-Concept Data to Care Study to Support Retention in Maternal HIV Treatment and Infant HIV Testing in Cape Town, South Africa

**DOI:** 10.1007/s10461-025-04726-7

**Published:** 2025-04-17

**Authors:** Tamsin K. Phillips, Yolanda Gomba, Pheposadi Mogoba, Florence Phelanyane, Kim Anderson, Benjamin H. Chi, Kate Clouse, Mary-Ann Davies, Jonathan Euvrard, Lucia Knight, Landon Myer, Elaine J. Abrams

**Affiliations:** 1 Division of Epidemiology & Biostatistics, School of Public Health, University of Cape Town, Cape Town, South Africa; 2 Centre for Infectious Disease Epidemiology & Research, School of Public Health, University of Cape Town, Cape Town, South Africa; 3 Department of Health and Wellness, Provincial Government of the Western Cape, Cape Town, South Africa; 4 Department of Obstetrics and Gynecology, School of Medicine, University of North Carolina at Chapel Hill, Chapel Hill, NC USA; 5 Vanderbilt University School of Nursing, Nashville, TN USA; 6 Vanderbilt Institute for Global Health, Nashville, TN USA; 7 Division of Social & Behavioral Sciences, School of Public Health, University of Cape Town, Cape Town, South Africa; 8 School of Public Health, University of the Western Cape, Bellville, South Africa; 9 Mailman School of Public Health, ICAP at Columbia University, Columbia University, New York, USA; 10 College of Physicians and Surgeons, Columbia University, New York, USA

**Keywords:** Data to care, Vertical HIV transmission prevention, Retention, Early infant diagnosis, South Africa

## Abstract

Data to Care (D2C) strategies– using routine data to identify and re-engage people living with HIV who are not in care– have shown promise in high-income settings but remain underexplored in lower-resource and vertical HIV transmission prevention (VTP) contexts. In this prospective, single-arm, proof-of-concept study, we used facility-linked public sector electronic medical records (Provincial Health Data Centre [PHDC] data) to identify, validate and trace postpartum VTP gaps among 336 mothers living with HIV and their infants in Cape Town, South Africa (March 2021–April 2022). Of 302 observed gaps, 123 (41%) were false, while 179 (59%) were probable gaps affecting 133 mother-infant pairs. Overall, 16% of mothers did not link to HIV care within 12 weeks postpartum and 13% had ART dispensing gaps, while 10% and 17% of infants had no HIV test around 10 weeks and six months, respectively. Only 100 (56%) probable gaps were confirmed through telephonic tracing, with 47 mothers subsequently re-linked to care. Mobility, stigma, and employment challenges contributed to disengagement. While our D2C approach streamlined tracing efforts, re-engagement remained difficult. Strategies integrating D2C approaches with interventions addressing social determinants are needed.

**Supplementary Information:**

The online version contains supplementary material available at 10.1007/s10461-025-04726-7.

## Introduction

Despite substantial progress towards the elimination of vertical transmission of HIV and impressive declines in new HIV infections in children worldwide, important gaps remain in early infant diagnosis and continuity of maternal antiretroviral therapy (ART) postpartum [1]. In South Africa, HIV transmission in the breastfeeding period has overtaken perinatal transmission with > 50% of new child infections attributed to breastfeeding [1]. Postpartum maternal ART interruptions and incomplete infant HIV testing at recommended time points are common [2–4]. These gaps in care may be due to individual, social and structural barriers including postpartum mobility and clinic transfers, younger maternal age and stigma [5–8].

Data to Care (D2C) is a collaborative public health strategy that supports the use of routine HIV surveillance data to facilitate the identification and linkage to care of people living with HIV who are not in care [9]. Experiences with D2C strategies have been predominantly focused on re-engagement of adults living with HIV in the United States (US), with encouraging evidence that employing D2C strategies within health departments can lead to improvements [10]. D2C programs in the US have highlighted the value of combining routine data sources to ensure a more real-world reflection of engagement in care and to assist with prioritizing scarce resources [11]; however, this approach has not been explored at scale in low- and middle-income countries (LMICs). To our knowledge, there has only been one published study of D2C strategies for people living with HIV in Africa: a pilot study in Mozambique that found when data from facility- and community-based services were combined to support children and adolescents living with HIV, improved retention and viral load testing uptake were observed [12].

D2C strategies may be particularly valuable to vertical HIV transmission prevention (VTP) programs. In South Africa and many other LMICs, mothers living with HIV and their infants move between locations of care to access VTP services over time [13]. Women may need to change clinic locations to receive ART and antenatal care during pregnancy, or they may be transferred out of integrated antenatal care and HIV services to routine ART clinics after delivery. These clinic transfers have been shown to present a risk for disengagement and poor outcomes [14, 15]. Routine transfers between clinics and individual geographic mobility also present a challenge for monitoring continuity of care, and ignoring transfers can result in large underestimates of retention [5, 16].

In the Western Cape (WC) province of South Africa, available public sector electronic health information systems, linked across facilities, are harmonized at the Provincial Health Data Centre (PHDC) to create an individual electronic medical record for patient care and health service management [17]. PHDC data were used extensively to support contact tracing and patient management during the COVID-19 pandemic [18, 19] and a strategy comparable to D2C has been used to support linkage between tertiary and primary care for patients diagnosed with tuberculosis [20]. In the Routine Electronic Mother-Infant Data (REMInD) study, we assessed (a) the validity of PHDC data to identify infants with incomplete HIV testing and mothers with ART interruptions after delivery, and (b) whether a proof-of-concept D2C strategy could be used to trace mother-infant pairs (MIPs) with gaps in VTP steps and facilitate re-engagement in care.

## Methods

### Local Context

This study focused on a large midwife obstetric unit (MOU) in Gugulethu, Cape Town, serving a catchment area of approximately 400 000 people [21]. The MOU provides perinatal care and HIV care to around 5 000 women annually and operates within a network of primary healthcare clinics offering antenatal care, child health care, and HIV services. In South Africa, all public-sector primary healthcare services are provided free of charge.

During pregnancy, women either begin antenatal care at the MOU or at a nearby primary healthcare clinic and then transfer to the MOU in the third trimester. Women already on ART transition into integrated antenatal and HIV care at the MOU. Deliveries occur at the MOU or a referral hospital if specialized care is needed. Postpartum, mothers and infants visit the MOU at 7–10 days, where mothers receive 2–3 months of ART before transferring to their nearest primary healthcare clinic for routine infant and HIV care. Infants receive HIV prophylaxis for 6–12 weeks based on perceived risk and start routine infant care, including HIV testing, at primary healthcare clinics from six weeks postpartum. Postnatal care may follow an integrated model, with mother and child seen together, or they may attend separate visits on different days or at different clinics, based on maternal preference and available services.

Within the Western Cape, patients accessing care in the public sector are allocated a unique patient identifier that links electronic records across public sector clinics through the PHDC. Data from the private sector and outside of the Western Cape are not linked. Robust systems, coordinated at the substructure level, leverage the PHDC data to monitor and trace new infant HIV diagnoses to facilitate linkage to treatment. However, identifying gaps in routine maternal ART or infant HIV testing relies on facility-specific missed appointment lists and patient tracing. If a mother or infant does not successfully transfer from the MOU to primary care or moves to a different clinic postpartum, the receiving clinic will not recognize them as missing until they have attended at least once and subsequently missed a scheduled visit.

### The REMInD Study Activities

**Fig. 1:**
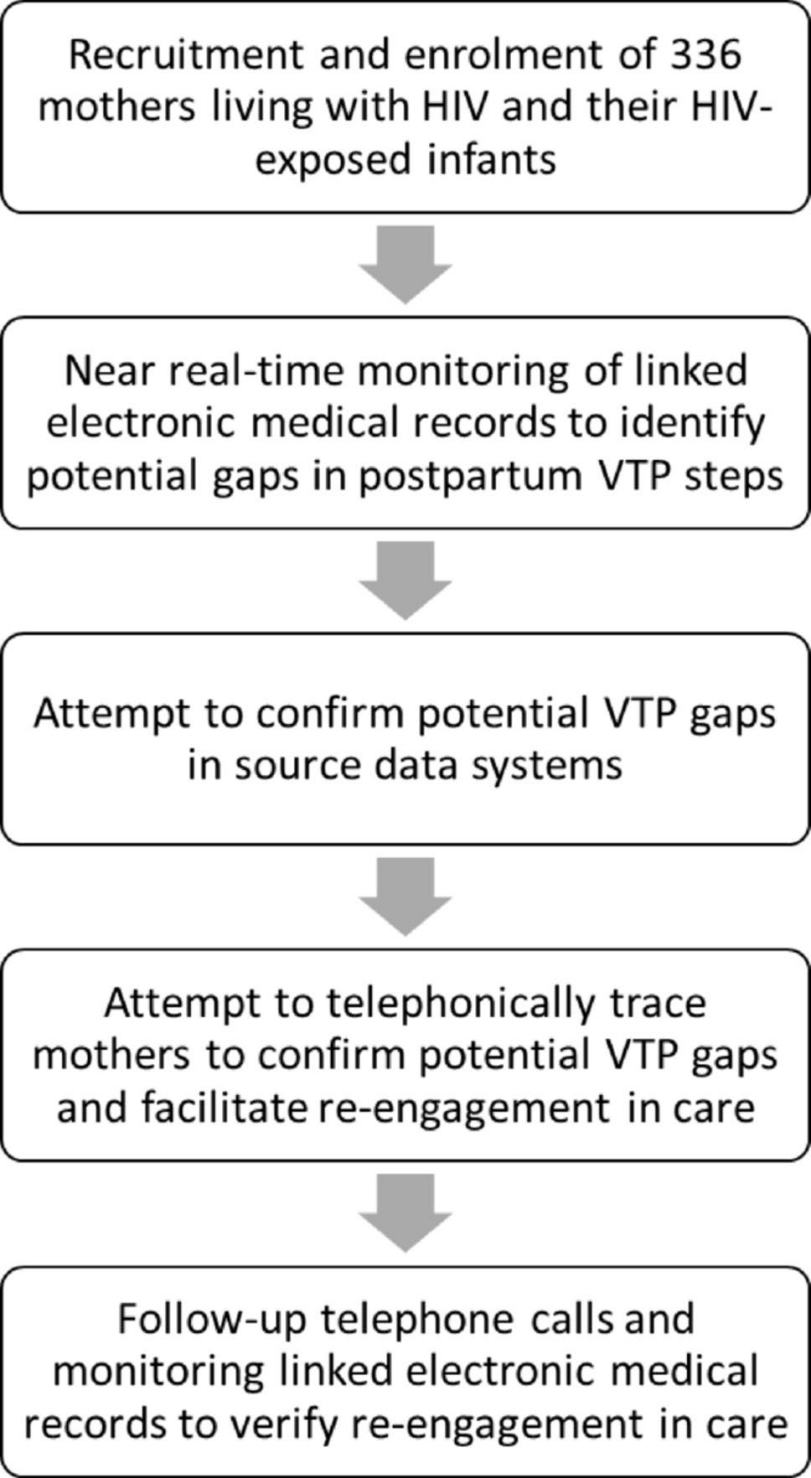
Summary of study activities. All activities in this proof-of-concept study were conducted by research staff (VTP– vertical HIV transmission prevention)

In a prospective cohort we (1) reviewed facility-linked public sector electronic medical records available in the PHDC in near real-time to observe gaps in key postpartum VTP steps, (2) attempted to confirm any observed gaps by reviewing data systems contributing to the PHDC, (3) attempted to telephonically contact participants to confirm care status and facilitate re-engagement in care for confirmed gaps, and (4) monitored successful re-engagement in care using PHDC data. Study activities are summarized in Fig. 1 and each component is described in more detail below.

### Study Recruitment and Enrolment

Pregnant and early postpartum women living with HIV who were attending antenatal care at the Gugulethu MOU between March 2021 and April 2022 were approached by a trained research fieldworker and screened for eligibility (age ≥ 18 years, in the third trimester or up to three weeks postpartum). Those who were eligible and willing to participate provided written informed consent, including consent to link to and monitor their and their infants’ electronic medical records, and to be traced by the study team should a gap in care be observed. At enrolment, women completed a comprehensive interviewer-administered structured questionnaire including socio-demographics and HIV treatment history. Contact details, unique health identifier and date of birth were recorded and stored separately from the questionnaire data to facilitate telephonic tracing and linkage to routine medical record data in the PHDC during study follow-up.

### Identifying Gaps in Postpartum VTP Steps

Using PHDC data, we actively monitored completion of key VTP steps (Table 1) in near real-time from enrolment through nine months postpartum. The selected steps, required for all mothers living with HIV and their HIV-exposed infants after transfer from the MOU to primary care [22], were considered most at risk of being overlooked by existing facility tracing due to facility transfers and geographic mobility. Study investigators reviewed the PHDC web-based individual patient record (Single Patient Viewer) and patient-level maternity and HIV cascade reports every 2–4 weeks to confirm expected care completion and identify potential gaps in VTP steps.

**Table 1:** Key steps in vertical HIV transmission prevention (VTP) examined in the remind study

Name	Definition^a^	Existing calculated indicator in the PHDC
Infant HIV testing around 10 weeks	Evidence of an HIV PCR test between 6 and 16 weeks of age	Yes
Infant HIV testing around 6 months	Evidence of an HIV PCR test between after 16 weeks and up to 36 weeks of age	Yes
Mother linked to primary HIV care after delivery	Evidence of ART dispensed at a primary care clinic after delivery and up to 12 weeks postpartum	No– new indicator generated using PHDC data
Mother continued ART after initial linkage to care	Following linkage to primary care, no gap of > 12 weeks between ART dispensing dates	No– new indicator generated using PHDC data
Infant with positive HIV PCR initiated and retained on ART	Evidence of ART initiation and continued dispensing of ART following infant HIV diagnosis	Yes

ART– antiretroviral therapy; PCR– polymerase chain reaction; PHDC– Provincial Health Data Centre

^a^Windows used in these definitions are based on the South African National Indicator Dataset [40]

### Validation of Potential Gaps in VTP Steps

Validation was conducted to identify false gaps (Table 2) in PHDC data. First, all missing infant HIV tests were cross-checked in the National Health Laboratory Services database to identify tests completed but not incorporated into the PHDC. Second, ART dispensing gaps were verified against the City of Cape Town health information system to ensure all data had been integrated into the PHDC. While the PHDC is administered by the WC Provincial Department of Health and Wellness, City of Cape Town clinics operate under local government administration using different data systems. Provincial and local government data systems are merged into the PHDC. No discrepancies were found in the City of Cape Town data over a period of ten months, leading to our discontinuation of this validation in December 2021.

**Table 2:** Categorization of gaps in vertical transmission prevention steps observed in the provincial health data centre (PHDC) following validation and patient tracing attempts

Gap category	Description
False gaps in VTP steps	All false gaps observed in the PHDC data due to data errors and unlinked data as defined below
Care received in WC	Care was received at a public sector clinic in the WC, but evidence of care access was not visible in the PHDC data. For example, an infant received an HIV test, but the test was not imported into the PHDC data.
Care received outside the WC	Participants contacted telephonically and reported that care was received outside of the WC. For example, a mother reported receiving ART from a clinic in another province. Data from clinics outside the WC is not included in the PHDC data.
Probable gaps in VTP steps	All confirmed and unconfirmed VTP gaps as defined below
Confirmed gap in VTP step	No evidence of accessing care could be found in the PHDC data or available source data. Participants contacted telephonically and confirmed that they had not accessed care and there was a gap in VTP steps.
Unconfirmed gap in VTP step	No evidence of accessing care could be found in the PHDC data or available source data. Participant could not be contacted telephonically so we were unable to confirm their care status.

PHDC data– Provincial Health Data Centre data (facility-linked public sector electronic health data); VTP– vertical HIV transmission prevention; WC– Western Cape

If evidence of completing a VTP step was missing, we conducted telephonic tracing of mothers to verify care engagement and confirm observed data gaps. This process identified cases where data were missing due to care received outside WC or data capture and health identifier issues within WC.

### Tracing and Linkage Back to Care

In this proof-of-concept study, all tracing activities were conducted by research staff, independent of routine clinic or community-based services. Once a VTP care gap was identified in PHDC data, details were shared with the study field team within a week, and tracing was initiated. A trained research fieldworker, functioning at the level of a lay community health worker, conducted tracing calls using contact details provided at enrolment, supplemented by routine medical records if needed. At least three attempts were made on different days and times. If successful, a structured questionnaire captured details of mother and infant care, and any missed VTP steps were recorded. If a VTP gap was confirmed, the fieldworker documented reasons (short open-ended questions), provided counseling, and facilitated re-engagement by issuing referral letters to the mother’s preferred clinic. Follow-up calls assessed whether care was resumed or if further support was needed. Re-engagement was also monitored via PHDC data.

### Data Analysis

Study data were captured into REDCap electronic data tools hosted at University of Cape Town and the data were exported for analysis in Stata 18 (StataCorp. 2023). To assess the validity of using existing PHDC data to identify infants with incomplete HIV testing and mothers with ART interruptions after delivery, we categorized all observed gaps in VTP steps as false or probable VTP gaps following validation and tracing (Table 2).

We described gaps in VTP steps using frequencies and proportions and described the proportion of infants diagnosed with HIV through nine months with an exact 95% confidence interval. We also explored the overlap of mothers who did not link to HIV care postpartum and infants with no infant HIV test at around 10 weeks (6–16 weeks), and mothers who had no ART dispensing and infants with no HIV test around six months (16–36 weeks) postpartum. These definitions sought to capture whether a mother was receiving ART in a window of time that mapped to expected infant HIV testing. In the case of a maternal or infant death in the study period, the mother or infant was still included in the denominator and not counted as having a gap in care after the time of death.

To assess whether a D2C strategy could be used to trace MIPs with gaps in VTP steps and facilitate re-engagement in care, we described the proportion of probable VTP gaps where we successfully contacted the mother telephonically, and the proportion of VTP gaps with telephonic contact that were successfully closed by linking the mother or infant back into care by the end of the nine-month follow-up period. Open-ended reasons for gaps in care collected during telephonic tracing were thematically coded into broad categories.

We described the enrolment characteristics of mothers with one, two or more, or no probable gaps in VTP steps (confirmed and unconfirmed gaps combined), including chi-squared tests and Wilcoxon rank sum tests for differences in characteristics between mothers with any versus no probable gaps. All statistical tests were two-sided, with α = 0.05. In supplementary analyses we also described characteristics of mothers with gaps in each VTP step.

Ethics.

This study was approved by the University of Cape Town Human Research Ethics Committee (HREC reference 513/2020). Participants with psychosocial concerns identified during the enrolment interviews or during tracing activities were referred to local support services at the Gugulethu Community Health Centre or nearby Non-Governmental Organizations for counselling and further assistance.

## Results

A total of 336 mothers were enrolled: 201 (60%) during the third trimester of pregnancy and 135 (40%) early postpartum (Table 3). Six mothers did not complete the enrolment questionnaire but were included in all other analyses. The median maternal age at delivery was 32 years (IQR 28–36, 14% were < 25 years old). Most of the cohort (85%) had started ART before the incident pregnancy (median six years since first ART initiation, interquartile range [IQR] 4–9 years) and 40% of these mothers reported that they had interrupted ART at least once before the pregnancy.

**Table 3:** Characteristics of mothers enrolled, comparing those with and without any probable gaps in maternal or infant vertical transmission prevention steps. Presented as N (%) unless specified

Characteristic	All women	No probable gaps	One probable gap	≥ 2 probable gaps	Any probable gaps	Test statistic^a^ (any vs. no gaps)	*p*-value (any vs. no gaps)
Total number enrolled	336	203	93	40	133		
Number with enrolment interview data	330	202	90	38	128		
Median maternal age at delivery, years, (IQR)	32 (28–36)	33 (29–37)	31 (28–35)	29 (25–33)	31 (27–35)	3.12	0.002
< 25 years	40 (12)	18 (9)	12 (13)	10 (26)	22 (17)	8.66	0.013
≥ 25, < 35 years	193 (58)	115 (57)	57 (63)	21 (55)	78 (61)		
≥ 35 years	97 (29)	69 (34)	21 (23)	7 (18)	28 (22)		
Completed high school	95 (29)	60 (30)	26 (29)	9 (24)	35 (27)	0.21	0.645
Currently employed	95 (28)	55 (27)	34 (38)	6 (16)	40 (31)	0.62	0.432
Receiving a government grant	241 (73)	145 (72)	69 (77)	27 (71)	96 (75)	0.41	0.521
Poverty tertiles (asset score and employment)							
Lowest score (most poverty)	121 (37)	70 (35)	34 (38)	17 (45)	51 (40)	2.57	0.277
Middle	110 (33)	74 (37)	20 (22)	16 (42)	36 (28)		
Highest (least poverty)	99 (30)	58 (29)	36 (40)	5 (13)	41 (32)		
Married/cohabiting	157 (48)	99 (49)	44 (49)	14 (37)	58 (45)	0.43	0.512
First pregnancy	36 (11)	20 (10)	10 (11)	6 (15)	16 (12)	0.40	0.528
Planned pregnancy	71 (22)	43 (21)	22 (24)	6 (16)	28 (22)	0.02	0.899
Newly diagnosed with HIV in this pregnancy	45 (14)	28 (14)	13 (14)	4 (11)	17 (13)	0.02	0.881
Disclosed to anyone	318 (96)	195 (97)	87 (97)	36 (95)	123 (96)	0.04	0.835
Disclosed to partner	246 (73)	155 (76)	65 (70)	26 (65)	91 (71)	1.31	0.252
Current regimen							
Tenofovir, Efavirenz, Emtricitabine	128 (39)	78 (39)	47 (52)	25 (66)	50 (39)	0.24	0.887
Tenofovir, Lamivudine, Dolutegravir	184 (56)	112 (55)	38 (42)	12 (32)	72 (56)		
Other	18 (5)	12 (6)	5 (6)	1 (3)	6 (5)		
ART history							
Started ART in this pregnancy	50 (15)	32 (16)	13 (14)	5 (13)	18 (14)	23.84	< 0.001
ART-experienced, ≥ 1 previous interruption	111 (34)	48 (24)	39 (43)	24 (63)	63 (49)		
ART-experienced, reports no previous interruptions	169 (51)	122 (60)	38 (42)	9 (24)	47 (37)		
Duration on ART before delivery, years (*n* = 280 with ART experience)	6.0 (3.9–9.0)	6.1 (3.9–9.4)	6.8 (4.3–9.2)	4.3 (2.8–6.5)	5.8 (3.4–8.6)	0.79	0.429

ART– antiretroviral therapy; IQR– interquartile range

^a^Chi-squared tests (χ^2^) were used for comparison of categorical variables and Wilcoxon rank-sum tests (W) for comparison of continuous variables

There were five infant deaths and no pregnancy losses or maternal deaths recorded during the study period. By nine months postpartum, five of 336 infants had been diagnosed with HIV (1.5% transmission; 95% CI 0.5–3.5; details in Supplemental Table 1). All five infants started ART immediately and remained in HIV care throughout the study period.

### Description of Gaps in VTP Steps

#### Completion of VTP Steps After Delivery Through Nine Months Postpartum

**Fig. 2:**
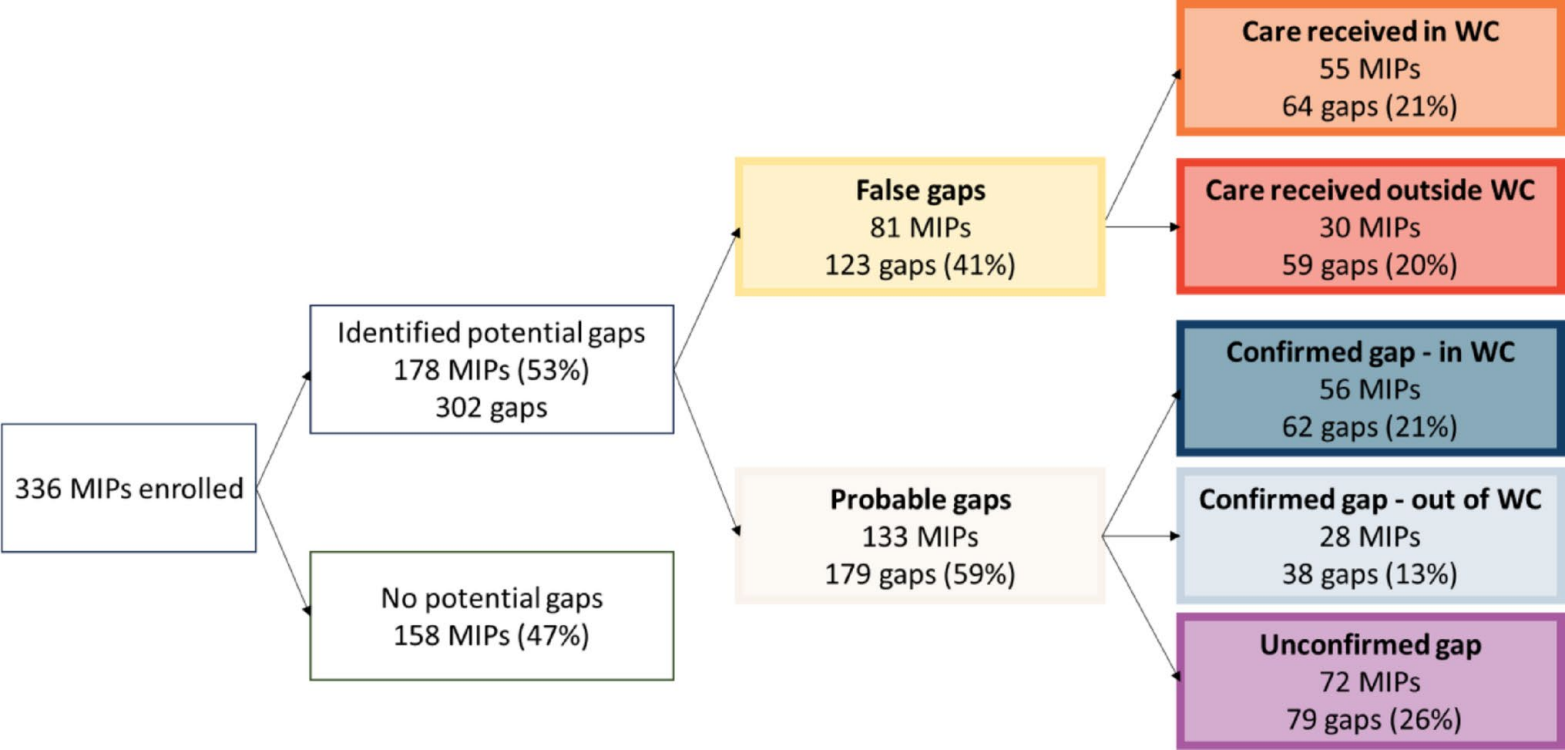
Classification of potential postpartum gaps identified in the Western Cape (WC) Provincial Health Data Centre among 336 mother infant pairs (MIPs)

**Fig. 3:**
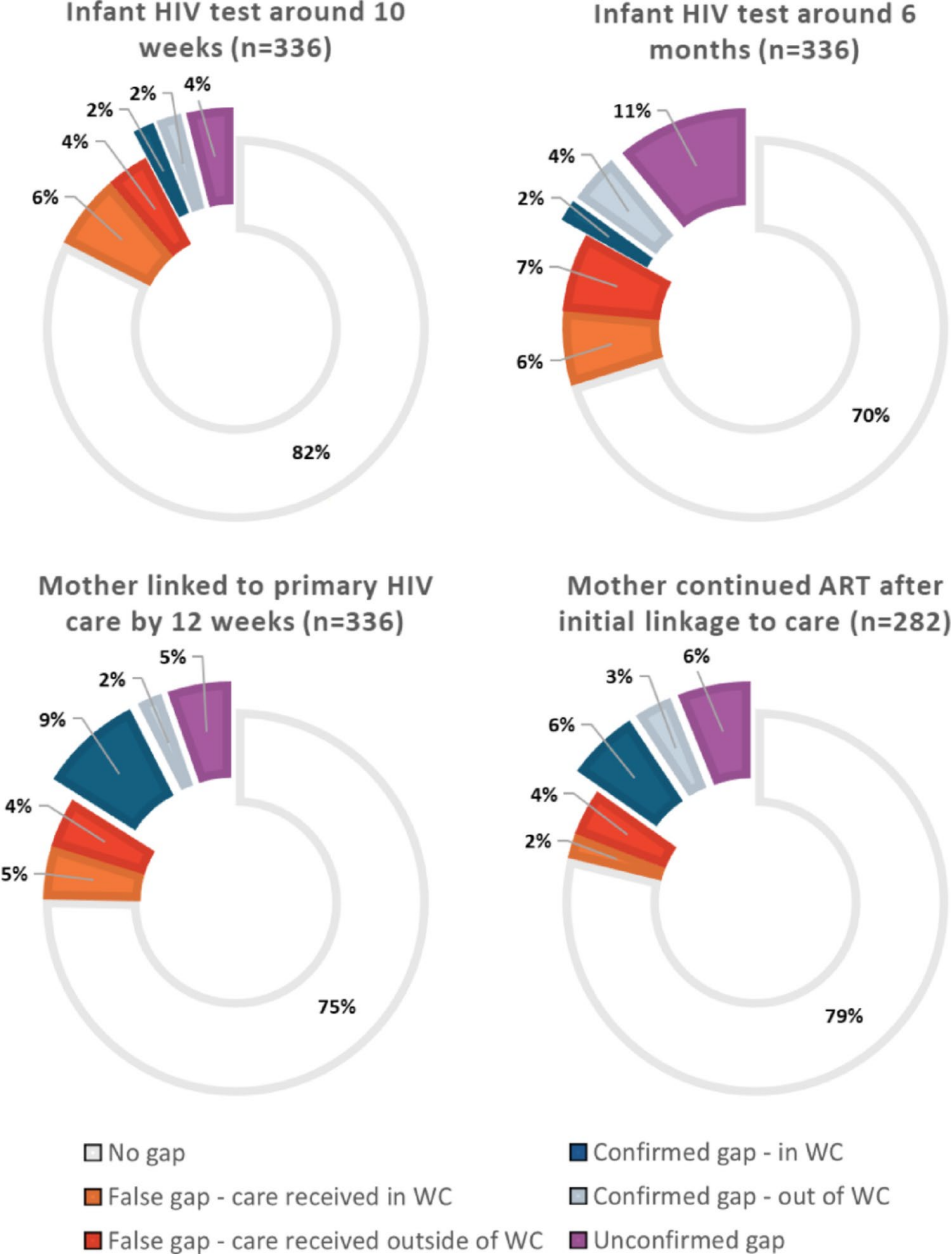
Classification of observed gaps in vertical transmission prevention steps identified in the Western Cape (WC) Provincial Health Data Centre among 336 mother infant pairs. ART - antiretroviral therapy

Overall, 158 MIPs (47%) had no observed gaps in VTP steps in the PHDC data. These MIPs had complete infant HIV testing at around 10 weeks and six months postpartum, the mother linked to care postpartum and had no gaps in ART dispensing up to nine months postpartum. There were 302 observed gaps in VTP steps in the remaining 178 MIPs when using PHDC data alone (Fig. 2). Through validation of laboratory data and telephone calls with participants, 123 gaps (41%) were found to be false: 64 (52% of false gaps) were determined to be due to missing PHDC data after care was received in the WC, and for 59 (48% of false gaps) care was accessed outside of the WC so the data were unavailable in the PHDC. The remaining 179 gaps (59%) were classified as probable: 100 (56% of probable gaps) were confirmed through telephonic contact and 79 (44% of probable gaps) were unconfirmed. The breakdown of gaps for each VTP step is shown in Fig. 3. Mothers with any (*n* = 133) versus no (*n* = 203) probable gaps had similar characteristics except for age and ART history prior to the pregnancy (Table 3). Mothers with any probable gap were slightly younger (17% versus 9% under 25 years, χ^2^ = 8.66, *p* = 0.013) and more likely to be ART-experienced with a history of a previous interruption before the pregnancy (49% versus 24%, χ^2^ = 23.84, *p* < 0.001), compared to mothers with no probable gaps in VTP steps. Among the 133 MIPs with any probable gap, 93 (70%) experienced only one gap, 34 (26%) experienced two gaps and six (5%) experienced three gaps; differences appeared to be heightened among mothers with two or more probable gaps (26% under 25 years and 63% ART-experienced with history of a previous interruption) compared to those with only one probably gap (13% under 25 years and 43% ART-experienced with history of a previous interruption). Characteristics of mothers with gaps in each of the key VTP steps are presented in Supplemental Table 2.

#### Data Feedback Loops

Throughout the study, investigators provided feedback on data errors to the PHDC to facilitate improvement over time. Among the 64 false gaps following care received in the WC, 42 (66%) were due to infant HIV tests not successfully importing from the National Health Laboratory Services into the PHDC. A large data import change was made in January 2022. The proportion of infants missing HIV tests due to data errors declined from 8.2% at 10 weeks (18/219) and 11.3% at 6 months (15/133) among infants with tests expected prior to February 2022, to 2.6% (3/117) and 2.9% (6/203) at 10 weeks and 6 months, respectively, among infants with HIV tests expected from February 2022 onwards. Other reasons for false gaps among participants accessing care in the WC included participants having multiple health identifiers that were not linked in the PHDC system (duplicate health identifiers were identified for 14 mothers), incorrect health identifier capture and, rarely, ART dispensing data not reflected in the PHDC data.

#### Success of D2C Strategy to Close Gaps in VTP Steps Between Delivery and Nine Months Postpartum

The combined confirmed and unconfirmed gaps in VTP steps following validation totaled 179 probable gaps from 133 MIPs (40% of 336) (Table 4). Among 336 enrolled mothers, 54 (16%) did not link to HIV care within 12 weeks of delivery and 43 (13%) linked to care after delivery but subsequently had a gap in ART dispensing by nine months postpartum. Combined, 29% of all mothers had a gap in ART. Among 336 infants, 25 (10%) and 57 (17%) did not have an HIV test at around 10 weeks or 6 months of age, respectively; 23% of all infants missed either HIV test.

**Table 4:** Summary of 179 probable gaps (including confirmed and unconfirmed) in vertical transmission prevention steps

	Number of probable gaps	Mother was successfully traced (gap confirmed)	Reported reasons for gap^a^	Successfully linked to care following tracing
All probable gaps	179 gaps (133 MIPs)	100 (56%) of probable gaps	63 (63%) of traced mothers	47 (47%) of those traced
Mother did not link to HIV care after delivery	54 MIPs 30% of all gaps 16% of mothers	37 (69%) of probable gaps	22 (59%) of traced mothers provided reasons: mobility Eastern Cape (7), transfer documentation challenges (8), work (5), mobility local (4), fear of stigma or being scolded (4), mental health (1), side effects (1), “no time” (1), death in family (1), transport money (1)	10 (27%) of those traced
Mother had gap of > 90 days with no ART dispensed after linking to care postpartum	43 MIPs 24% of all gaps 13% of mothers	29 (67%) of probable gaps	21 (72%) of traced mothers provided reasons: mobility Eastern Cape (10), mobility local (6), transfer documentation challenges (5), fear of stigma or being scolded (3), work (2), alcohol use (1), tired of attending (1)	19 (66%) of those traced
No infant 10-week HIV test	26 MIPs 14% of all gaps 10% of infants	12 (46%) of probable gaps	7 (58%) of traced mothers provided reasons: mobility Eastern Cape (4), mobility Gauteng (1), transfer documentation challenges (1), tired of attending (1)	10 (83%) of those traced
No infant 6-month HIV test	56 MIPs 32% of all gaps 17% of infants	22 (39%) of probable gaps	13 (59%) of traced mothers provided reasons: mobility Eastern Cape (13), negative 10-week result (1)	8 (36%) of those traced

ART– antiretroviral therapy; MIP– mother-infant pair

^a^Number of women reporting the reason in brackets. Reasons were reported in brief open-ended questions asked during a structured telephone call. Multiple reasons could be reported per gap

Only 100 of 179 mothers (56%) with probable gaps were successfully contacted telephonically and confirmed (Table 4). Of the 100 successfully traced, 47 gaps (47%) were subsequently closed with evidence of the mother or infant successfully relinking to care. A higher proportion of successful re-linkage to care was observed for the 10-week infant HIV test (83% of those contacted linked to care) and later postpartum ART gaps (66% of those contacted linked to care), compared to the 6-month infant HIV test (36% of those contacted linked to care) and mother linking to care after delivery (27% of those contacted linked to care). Among the 79 unconfirmed gaps, the main challenge was calls not being answered or going directly to voicemail (*n* = 53) and some were answered but reported to be incorrect numbers (*n* = 5). Some calls were answered by the participant who was unable to speak freely due to privacy concerns (*n* = 6). Four gaps were not traced as there was evidence of return to care prior to tracing, and for 12 gaps the reason for failed tracing was not recorded.

Reasons for not connecting to care were provided by mothers for 63 gaps (63% of the 100 successfully traced, Table 4). The most common reasons provided were related to geographic mobility and clinic transfer documentation challenges both within and outside of the WC. This included being turned away for not having documentation of treatment history, difficulty getting a transfer letter after moving out of the WC, and fear of not being served if they presented without transfer documentation. Other common reasons were fear of stigma or being scolded at the clinic and employment-related challenges.

#### Overlap of Mother and Infant Gaps in VTP Steps

Little overlap was observed between gaps in VTP steps among mothers and their infants with only one mother and infant both being out of care at around 10 weeks and five both being out of care at around six months (Table 5). There were 53 MIPs (16%) where the mother had not linked to HIV care postpartum even though the infant had received a 10-week HIV test, and there were 10 MIPs (3%) where the infant had not completed a 10-week test, but the mother had linked to HIV care postpartum. By nine months postpartum, there were 41 MIPs (12%) where the mother was not accessing HIV care, but the infant had received a six-month HIV test, and there were 53 MIPs (16%) where the infant had no six-month test while the mother was engaged in HIV care.

**Table 5:** Overlap of mother and infant gaps in care. Presented as N (%) out of 335 mother-infant pairs (MIPs) at 10 weeks and 332 MIPs at six months postpartum. (Numbers exclude infant deaths: one death before 10 weeks and three deaths between 10 weeks and 6 months postpartum)

	Mother linked to care postpartum	Mother did not link to care postpartum
Infant 10-week HIV test done	271 (81)	53 (16)
No infant 10-week HIV test	10 (3)	1 (< 1)

	Mother in care around 6 months	Mother not in care around 6 months
Infant 6-month HIV test done	223 (67)	41 (12)
No infant 6-month HIV test	53 (16)	15 (5)

## Discussion

In this study, we successfully used existing electronic medical record data (linked across public sector data sources and clinics) to screen out MIPs who had successfully completed key VTP steps within the WC, with close to 50% of the cohort completing all steps and requiring no further action. Lack of access to health data from outside the WC along with data import challenges and duplicate health identifiers in the WC resulted in some misclassification of gaps. Tracing MIPs with gaps in VTP steps and facilitating re-engagement in care in this low-resource setting in Cape Town, South Africa, proved challenging. Reassuringly, we observed low HIV transmission through nine months postpartum; however, we observed an ongoing need to strengthen postpartum ART continuity and infant HIV test completion. Our findings highlight the potential value and important challenges implementing a D2C strategy to identify and trace MIPs with gaps in VTP steps in this setting.

These results confirm the value of combining routine HIV data sources and using unique patient identifiers to link across health facilities, to obtain a more realistic view of engagement in HIV care and thus support more efficient patient tracing [11]. Geographic mobility in this study contributed both to misclassification of gaps and to reasons for gaps in VTP steps. In settings such as ours where mobility is common, improved health service adaptability is needed to ensure both patients’ continued engagement in care and continued provision of services despite changes in care location. Strategies to facilitate seamless clinic transfers are also urgently needed as this remains a substantial barrier, despite guidelines stating that people cannot be turned away from HIV care without transfer documentation [23]. While routine electronic data sources are not without challenges [24–27], the combination of multiple data sources linked across facilities allowed us to minimize missing data due to mobility, thereby minimizing the number of people misclassified as out of care. Harmonized data from multiple sources may not be available in all LMIC settings, or even HICs; however, it is still possible to implement similar strategies. A study implementing a D2C strategy in Mozambique, for example, held regular joint data reviews to compare and share facility and community data to identify children and adolescents who were out of HIV care [12]. Similarly, the CoRECT trial in the US held monthly meetings to consolidate lists of people out of care based on laboratory surveillance data and clinic appointment data [28, 29]. In both studies, joint reviews created an opportunity for collaboration between partners and for decisions to be made around appropriate next steps and support interventions. In Mozambique, such collaboration meant support services and linkage interventions could be moved from facilities to community-based services, allowing for greater geographic coverage and accommodation of mobility and movement between clinics [12].

Despite the value of a D2C approach, further research is needed to establish robust evidence for D2C strategies in LMIC settings. Our study highlights that identifying individuals with gaps in VTP care is only the first step– ensuring continuity of care remains a major challenge. Even with a dedicated research team, contacting mothers and facilitating re-engagement was challenging. Effective D2C strategies must integrate the strengths of routine HIV surveillance data with behavioral interventions to optimize relinkage and retention. Although all women in our study provided written consent for record review and tracing, telephonic tracing was only successful for 56% of MIPs with probable gaps. Among mothers who were contacted, fear of stigma or scolding at the clinic were key reasons given for not connecting to care, which may also have influenced tracing success. Compassionate, patient-centered care has been linked to better retention in HIV care in South Africa [30]. Strong, bidirectional trust between patients, providers, and the broader health system, is essential for improving health system performance and patient-centered care [31]. Without this foundation of trust, the impact of D2C strategies on service delivery and health outcomes may be limited. Additionally, mechanisms must be in place to ensure patients understand how their routine health data is used, with appropriate consent processes in routine health settings [32].

It is well documented that barriers to engagement in HIV care are often related to broader social and structural factors not directly linked to HIV [33]. Mothers living with HIV, including those who interrupt HIV care postpartum or who do not complete HIV testing visits with their infants, are often living in unstable circumstances and need social support services in addition to HIV care [34, 35]. Although integration of care for MIPs is strongly recommended in the national ART guidelines [23], implementation remains complex [36]. It is not uncommon for infants in our setting to live apart from their mothers, with other primary caregivers [37], and after delivery women may not be identified as postpartum in routine HIV services. Challenges with mobility and clinic transfers in this cohort, as well as our finding of missed opportunities where mother and infant gaps in care do not overlap, may reflect some level of housing and economic instability during this vulnerable early postpartum period. Patients who received a D2C intervention in the US reported how the program helped to identify needs for social assistance as well as assisting with difficulties navigating HIV care [38]. Similarly, US providers felt that identifying and addressing social and structural barriers was often essential to enable re-engagement in HIV care and was a critical function of the D2C team [39]. Tracing efforts therefore need to be integrated with community-based services and incorporate links to the required social support. It is also important to note that, while targeted tracing such as through D2C strategies can be useful to ensure short-term completion of care, there is a clear and urgent need for broader structural interventions to reduce the overall vulnerability in this population. A randomized D2C trial in the US found that although the D2C approach improved initial re-engagement in HIV care, it did not impact durable viral suppression [28]. In the context of large-scale investments made by international and national donors to improve data systems—alongside shrinking health budgets and growing program need—a D2C strategy should be one piece of a multicomponent approach to efficiently ensure completion of VTP steps, while also addressing broader social and structural barriers to improve health for all vulnerable mothers and their infants.

This work should be interpreted with the following limitations in mind. First, while the routine electronic medical record data in the WC links numerous data sources and uses a unique patient identifier to link individual data between facilities, this does not extend outside of the WC or to private sector health services. In previous research in this setting, we have documented mobility outside of the WC in 9% of mothers in the first two years postpartum [5]. While this is a limitation of the data, having these individuals on the list of patients out of care is a potentially important opportunity to ensure continuity of care. Our results highlight that connecting to infant HIV testing services and continued ART when traveling outside of the WC is a substantial challenge and additional support and improved data systems are necessary to facilitate and track interprovincial transfers. Second, this study recruited women accessing antenatal care at a single clinic in Cape Town, the D2C strategy was implemented by a research team and the study made use of a unique harmonized data system in the WC. While this limits the generalizability of the findings, the lessons learnt from this study are likely to apply to many high-HIV-burden settings on the continent where mobility is common and completion of VTP steps remains a challenge. There are continued efforts to implement electronic health records in many LMICs and electronic health record systems do exist in other African countries. The data sources incorporated in the WC electronic health data are largely available in the rest of South Africa and, along with a unique health identifier, mean that D2C strategies such as that presented in this study should be possible. Further research is needed to understand adaptations needed to incorporate tracing activities into routine health services and how best to tailor implementation strategies to specific contexts and their available data systems and resources. Finally, we observed low rates of infant HIV transmission in this study; however, follow-up was limited to six months and breastfeeding status is not recorded in PHDC data, so we were unable to ascertain final infant HIV outcomes. The short follow-up duration also means we do not know the longer-term patterns of maternal HIV care engagement and we did not measure maternal viral load which will be an important indicator for future studies. HIV transmission through breastfeeding is an important contributor to new infant HIV infections and the incomplete infant HIV testing and maternal retention observed in this cohort highlights the need for ongoing investment in interventions to support postpartum retention in care.

In conclusion, in this proof-of-concept study of a D2C strategy to strengthen completion of VTP steps in South Africa, facility-linked electronic data provided a more realistic view of VTP completion and allowed for targeted tracing of MIPs with probable gaps. Strategies that foster mutual trust between patients, providers, and the health system—and strengthen our ability to reconnect with those out of care—are urgently needed. Future research should consider adaptations needed to embed D2C strategies into routine health services in LMIC settings, and how to incorporate the strengths of D2C with links to community-based services and structural interventions that speak to the broader needs of women living with HIV and their children.

## Supplementary Information

Below is the link to the electronic supplementary material.

Supplementary material 1 (DOCX 24.5 kb)

## Abbreviations

ART: Antiretroviral therapy
D2C: Data to care
LMIC: Low- and middle-income countries
MIP: Mother-infant pair
MOU: Midwife obstetric unit
PCR: Polymerase chain reaction
PHDC: Provincial health data centre
WC: Western cape
