# Supplementary material for: Routine Electronic Mother-Infant Data (REMInD): A proof-of-Concept Data to Care Study to Support Retention in Maternal HIV Treatment and Infant HIV Testing in Cape Town, South Africa
Source: AIDS Behav. 2025 Apr 17;29(9):2689–700. doi: 10.1007/s10461-025-04726-7 (PMC12432080; doi:10.1007/s10461-025-04726-7)
Supplement: Supplementary file 1 — Supplementary material 1 (DOCX 24.5 kb) [file 10461_2025_4726_MOESM1_ESM.docx]

**Supplemental inserts**

Supplementary Table 1. Five vertical transmissions recorded during the study up to the 6-month polymerase chain reaction (PCR) test

| Time of infant HIV test^a^ | Likely timing of transmission | Mother’s HIV care in pregnancy^b^ | Mother’s HIV care postpartum^b^ |
| --- | --- | --- | --- |
| Birth PCR positive | In-utero/ intrapartum | Mother on ART pre-pregnancy  Regimen: tenofovir-lamivudine-dolutegravir  Self-reported adherence challenges during pregnancy^c^  VL >1000 copies/mL in pregnancy  VL >1000 copies/mL at delivery | Continued ART postpartum  VL <50 copies/mL four months postpartum |
| Indeterminate at birth  Positive at 10 weeks | In-utero/ intrapartum | Mother on ART pre-pregnancy  Regimen: dolutegravir, emtricitabine, zidovudine  VL >1000 copies/mL in pregnancy  VL <50 copies/mL at delivery | Continued ART postpartum  VL 50-1000 copies/mL nine months postpartum |
| Negative at birth  Positive at 10 weeks | In-utero/ intrapartum | Mother on ART pre-pregnancy  Regimen: tenofovir-emtricitabine-efavirenz  Gap in ART in PHDC data during pregnancy  VL >1000 copies/mL in pregnancy  VL >1000 copies/mL at delivery | No ART postpartum  No VL after delivery |
| Indeterminate at birth  Repeat test positive  Second repeat test negative (all within 7 days postpartum)  Positive at 10 weeks | In-utero/ intrapartum | Mother on ART pre-pregnancy  Regimen: tenofovir-lamivudine-dolutegravir  Gap in ART before entering antenatal care (after estimated conception date)  No VL available during pregnancy  VL <50 copies/mL at delivery | Continued ART postpartum  No VL after delivery |
| Negative at birth  Missed 10-week PCR  Positive at 6 months | Breastfeeding | Mother diagnosed in pregnancy  Regimen: tenofovir-lamivudine-dolutegravir from ~3 months prior to delivery  VL <50 copies/mL at delivery | No ART postpartum  No VL after delivery |

ART – antiretroviral therapy; PCR – polymerase chain reaction; PHDC – Provincial Health Data Centre; VL – viral load

^a^This reflects transmission diagnosed through to the 6-month infant HIV PCR test and does not reflect full breastfeeding transmissions as transmissions after the end of the study period (nine months postpartum) are not known.

^b^Mother’s HIV care in pregnancy and postpartum was ascertained from the PHDC data.

^c^Self-reported adherence was assessed during the study enrolment questionnaire.

Supplemental table 2. Characteristics of mothers enrolled, comparing those with and without any probable gaps in vertical transmission prevention steps. Presented as n (%) unless specified.

| Characteristic | All women | Any probable gaps | Gaps in key VTP steps contributing to any probable gaps ^a^ | | | | No probable gaps |
| --- | --- | --- | --- | --- | --- | --- | --- |
|  |  |  | Not linked to HIV care postpartum | Gap of >90 days with no ART dispensed | No 10-week infant HIV test | No 6-month infant HIV test |  |
| Total number enrolled | 336 | 133 | 54 | 43 | 26 | 56 | 203 |
| Number with enrolment interview data | 330 | 128 | 51 | 42 | 25 | 53 | 202 |
| Median maternal age at delivery, years, (IQR) | 32 (28-36) | 31 (27-35) | 30 (25-35) | 30 (26-34) | 29 (26-34) | 32 (27-35) | 33 (29-37) |
| <25 years | 40 (12) | 22 (17) | 13 (25) | 7 (17) | 6 (24) | 8 (15) | 18 (9) |
| ≥25, <35 years | 193 (58) | 78 (61) | 28 (55) | 28 (68) | 13 (52) | 32 (60) | 115 (57) |
| ≥35 years | 97 (29) | 28 (22) | 10 (20) | 6 (15) | 6 (24) | 13 (25) | 69 (34) |
| Completed high school | 95 (29) | 35 (27) | 16 (31) | 9 (21) | 7 (28) | 13 (25) | 60 (30) |
| Currently employed | 95 (28) | 40 (30) | 12 (24) | 9 (21) | 10 (40) | 18 (34) | 55 (27) |
| Receiving a government grant | 241 (73) | 96 (75) | 39 (76) | 31 (74) | 18 (72) | 38 (72) | 145 (72) |
| Poverty tertiles (asset score and employment) |  |  |  |  |  |  |  |
| Lowest score (most poverty) | 121 (37) | 51 (40) | 20 (39) | 18 (43) | 13 (52) | 19 (36) | 70 (35) |
| Middle | 110 (33) | 36 (28) | 15 (29) | 16 (38) | 6 (24) | 17 (32) | 74 (37) |
| Highest (least poverty) | 99 (30) | 41 (32) | 16 (31) | 8 (19) | 6 (24) | 17 (32) | 58 (29) |
| Married/cohabiting | 157 (48) | 58 (45) | 20 (39) | 15 (36) | 12 (48) | 27 (51) | 99 (49) |
| First pregnancy | 36 (11) | 16 (12) | 7 (13) | 8 (19) | 3 (12) | 5 (9) | 20 (10) |
| Planned pregnancy | 71 (22) | 28 (22) | 10 (20) | 7 (17) | 6 (24) | 12 (23) | 43 (21) |
| Newly diagnosed with HIV in this pregnancy | 45 (14) | 17 (13) | 7 (14) | 6 (14) | 2 (8) | 7 (13) | 28 (14) |
| Disclosed to anyone | 318 (96) | 123 (96) | 48 (94) | 40 (95) | 24 (96) | 21 (96) | 195 (97) |
| Disclosed to partner | 246 (73) | 91 (74) | 33 (69) | 31 (72) | 18 (69) | 37 (66) | 155 (79) |
| Current regimen |  |  |  |  |  |  |  |
| Tenofovir, Efavirenz, Emtricitabine | 128 (39) | 50 (39) | 31 (61) | 24 (57) | 16 (64) | 30 (57) | 78 (39) |
| Tenofovir, Lamivudine, Dolutegravir | 184 (56) | 72 (56) | 19 (37) | 18 (43) | 14 (56) | 19 (36) | 112 (55) |
| Other | 18 (5) | 6 (5) | 1 (2) | 0 | 2 (8) | 4 (8) | 12 (6) |
| ART history |  |  |  |  |  |  |  |
| Started ART in this pregnancy | 50 (15) | 18 (14) | 6 (12) | 8 (19) | 3 (12) | 8 (15) | 32 (16) |
| ART-experienced, ≥1 previous interruption | 111 (34) | 63 (49) | 32 (63) | 20 (48) | 14 (56) | 23 (43) | 48 (24) |
| ART-experienced, reports no previous interruptions | 169 (51) | 47 (37) | 13 (25) | 14 (33) | 8 (32) | 22 (42) | 122 (60) |
| Duration on ART before delivery, years (n=280 with ART experience) | 6.0 (3.9-9.0) | 5.8 (3.4-8.6) | 5.6 (4.1-7.3) | 5.0 (3.1-7.6) | 5.2 (3.0-9.2) | 5.6 (3.1-9.3) | 6.1 (3.9-9.4) |

ART – antiretroviral therapy; IQR – interquartile range; VTP – vertical transmission prevention

^a^ Numbers for each key gap do not sum to total for any probable gaps as participants may have had gaps in multiple steps.
